# Seroprevalence of HCV infection among Indian patients with hemodialysis

**DOI:** 10.6026/973206300200868

**Published:** 2024-08-31

**Authors:** Kamalraj Mohan, Subha Vajjiravel Jagannath, Ambuja Sekhar, Akila Krishnamoorthy, Anitha Srinivasagalu, Sijimol Shanmugam, Senthamarai Srinivasan, Sivasankari Selvaraj

**Affiliations:** 1 Department of Microbiology, Meenakshi Medical College Hospital & Research Institute, Meenakshi Academy of Higher Education and Research (Deemed to be University), Kanchipuram-631552, Tamilnadu, India;

**Keywords:** HCV infection, RT-PCR, hemodialysis

## Abstract

Hepatitis C virus (HCV) exists as the most common hepatotropic disease with a severe complication in significant patients. Maximum incidence of HCV infection occurred specifically in hemodialysis patients and they were related with increased illness and fatality rate. Therefore, it is of interest to know the seroprevalence of HCV infections in hemodialysis patients using anti-HCV and PCR diagnosis. Hence, this study was carried out at a tertiary care hospital for a period of 12 months. 92 blood samples were collected and subjected for the detection of HCV antibodies using ELISA followed by RT-PCR. Out of 92 HD patient samples, 7 were positive for Anti-HCV and 9 samples showed positive for HCV RNA. Thus, ELISA has less sensitivity with false negative rate for identification of HCV infection. Thus, hemodialysis patients should be routinely screened for HCV using RT-PCR for adequate management of the disease.

## Background:

Hepatitis virus is the most predominant in causing inflammation of the liver. Hepatitis C (HCV) categorized under the Flaviviridae family, which is enveloped, positive-sense single-stranded RNA. HCV genomic RNA comprises a structural and non-structural genetic characterization. Thus HCV consist of seven genotypes besides 90 subtypes were geographically distributed in diverse patterns [1]. Classification of Hepatitis includes A, B, C, D and E. Among these, Hepatitis C virus was known to be the most predominant one in blood borne infection, which mainly affects the patients on (MHD) maintenance hemodialysis. Significance of the HCV is a major public health concern in several countries. Globally, every year approximately 1.5 million were infected with HCV infection, of which 58 million people were existed with chronic HCV infection [2]. As per World Health Organization (WHO) guidelines, HCV screening in blood donors indicates the lowest detection rate for HCV identification in infected cases when comparing with other countries [3]. However, the occurrence rate of infection is around 6-60% in HD individuals, whereas few studies in India vary from 4.3% to 45%. Moreover, various risk factors were recognized for the prevalence of HCV infected HD patients, such as frequent blood transfusion, duration of HD treatment, and also by nosocomial transmissions. Henceforth, inappropriate infection-control measures may be prone for chronic hepatitis & carcinoma [4, 5]. Anti-HCV screening must be done consistently in patients due to the probability of HCV infection according to CDC (Centre of Disease Control) recommendations. Almost all the laboratories were depending upon Anti-HCV detection, but these may fail to detect the acute phase of the disease, exclusively in HD patients. Most probably antibody screening method gives false positive in few cases. To avoid false - negative antibody detection method, alternatively recommended was HCV RNA PCR method. They are the gold standard & confirmatory method for screening HCV even at occult infection. Viral load will be monitored by the RT- PCR for the HCV infected patients [5]. HCV core Ag is another method for identification of HCV infected patients at the earlier stage. Predominantly in India HCV among HD patients have been done with antibody detection methods. Highest prevalent rate of HCV in dialysis unit may evolve to severe complications [10-11]. Therefore, it is of interest to find out the prevalence of HCV among HD patients by ELISA and RT-PCR methods.

## Materials & Methods:

The Department of Microbiology at Meenakshi Medical College Hospital & Research Institute (MMCH&RI), Kanchipuram, India, conducted a prospective study over a 12-month period from March 2023 to February 2024. The Institutional Ethics Committee (IEC) [IEC NO: MMCHRI IEC/18/23] approved the study and obtained informed consent from all participants. Inclusion criteria focused on hemodialysis (HD) patients undergoing frequent blood transfusions, encompassing all age groups, to assess HCV prevalence across different demographics. We also included patients who had a documented history of HCV exposure or past infection to assess the effectiveness of the diagnostic. Ethical standards and the integrity of the study were protected by excluding patients who did not want to participate, had other hepatotropic viruses like Hepatitis B virus (HBV) or HIV, or had severe comorbidities that could get in the way of study procedures. We also excluded patients who had previously received antiviral treatment for HCV. We collected 5 ml of blood from each patient under aseptic precautions, separated the serum, and stored it at -20°C for further analysis. The study utilized ELISA for detecting anti-HCV antibodies and RT-PCR for identifying HCV RNA. As part of the data analysis, seroprevalence rates were calculated and the diagnostic performance of ELISA and RT-PCR methods were compared to see which one was better at finding HCV infections in hemodialysis patients. This design allowed for a comprehensive assessment of HCV prevalence and the reliability of diagnostic tests within this high-risk group.

## ELISA:

J. Mitra HCV Microlisa Kit (India) was used to detect anti-HCV antibodies. Initially, serum samples were diluted 1:100 with sample diluent (10 µL of serum mixed with 1 mL of sample diluent) and vortexed. Next, 100 µL of the diluted samples and calibrators were added to the wells of the ELISA plate. We then covered the plate and incubated it at 37°C for 30 minutes. After incubation, we washed the wells six times with the working wash solution. Subsequently, 100 µL of conjugate was added to each well, and the plate was covered and incubated again at 37°C for 30 minutes. After a second wash cycle of six times with the working wash solution, 100 µL of working substrate solution was added to each well, and the plate was incubated in the dark at room temperature (20-30°C) for 30 minutes. The reaction was stopped by adding 50 µL of stop solution, and the absorbance was read at 450 nm/630 nm using a bio chromatic reader within 30 minutes [5, 7].

## RNA extraction:

Extraction was performed by using Helini Biomolecules RNA Extraction Kit & followed as per the manufacturer's protocol. Briefly, 200 µL of patient serum was mixed with 600 µL of lysis buffer and incubated at room temperature for 10 minutes. After incubation, 600 µL of ethanol was added, and the mixture was transferred to a spin column. We centrifuged the column at 12,000 rpm for 1 minute and discarded the flow-through. The column was washed with 500 µL of wash buffer I and centrifuged at 12,000 rpm for 1 minute, followed by a second wash with 500 µL of wash buffer II and centrifugation at 12,000 rpm for 1 minute. Finally, RNA was eluted with 50 µL of RNase-free water and stored at -20°C until further use [7].

## RT-PCR

We used the Qiagen Roto-Gene instrument to subject the extracted RNA to real-time polymerase chain reaction (RT-PCR). The RT-PCR protocol included the following thermal cycling conditions: an initial denaturation step at 90°C for 20 seconds, followed by annealing at 56°C for 20 seconds, and extension at 56°C for 20 seconds. We repeated this cycle for a total of 45 cycles. We monitored the successful amplification in real-time, and the generated amplification graph at the end of the cycles indicated the presence of HCV RNA [7].

## Statistical analysis:

The data obtained from the ELISA and RT-PCR tests were analyzed using descriptive statistics to determine the seroprevalence of HCV infection among hemodialysis patients. The number and percentage of patients testing positive for Anti-HCV antibodies and HCV RNA were calculated. The overall seroprevalence of HCV infection among the hemodialysis patients was determined by the proportion of positive cases identified by RT-PCR out of the total samples tested [7].

## Result & Discussion:

Distribution of age factor in hemodialysis patients reveals that the largest proportion accounting for 46% & the overall patient population is within the age of 41-60 years. This is charted by patients aged 21-40 years, who make up 32% of the population. Patient exists at an age of 61 & 80 outlined up to 22% of the group, whilst those who are 80 years or older make up a very small part, at 1.08%. In total, the study included 92 patients, with each age group accounting for a portion of the total, resulting in a cumulative percentage of 100% (Table 1). Out of the total of 70 male hemodialysis patients, 7 tested positive for HCV, leading to a positivity rate of 14%. In contrast, out of the 22 female patients tested, only 2 tested positive, resulting in a lower positivity rate of 9%. Out of the total group of patients, 9 out of 92 were found to be positive for HCV, resulting in an overall positivity rate of 9.8% (Table 2). The frequency of HCV in hemodialysis patients was further examined using different diagnostic techniques. Out of the 92 patients that were examined, 7 of them were found to have anti-HCV antibodies, indicating a seroprevalence rate of 7.6%. In addition, 9 patients were found to have tested positive for HCV RNA, leading to a seroprevalence rate of 9.8%. This indicates a significantly increased occurrence of active HCV infection in comparison to solely detecting HCV antibodies (Table 3).

Hepatitis C virus is considered as a major global anxiety with an increased risk of numerous complications includes hepatic cirrhosis & hepatocellular carcinoma. However, in hemodialysis (HD) patients, these HCV infections are related to various systemic illnesses to renal disease and cardiovascular events. Henceforth, the acquisition of infected patients, were recognized as persistent of renal failure due to recurrent hemodialysis [2-3]. Most of the studies have documented that HD patients infected with HCV is because of nosocomial transmission & unclean equipment are additional source of risk factors for acquiring Hepatitis C [12]. Since, few studies revealed that the highest anti-HCV positivity rate of 24-26% cases was seen in some patients [6]. Another study has reported % 27.7 in the year of 2009 by Jamil *et al.* Similar studies done by Chadra *et al.* was accounted for nearly 46%.8 which coincides with the study done by Agarwal *et al.* [4]. A wide geographical variation was observed in hemodialysis patients, a relative HCV infection and renal disorders also recognized [5]. Prevalence rate of anti-HCV patients with hemodialysis were at the range of 12-13% are reported previously, few studies showed 6.9%, & 2.7 % recently [8]. In our study 9.8% HCV prevalence was seen in the HD patients who coincides with the previous studies 8 % by Madhavan *et al.* [7]. This study comprises the extreme number of HCV positive patients were seen between 41-60 (46%) age group, which coincides with the previous conducted by Nivetha *et al.*, at 2022 [9]. Studies found that the maximum frequency rate of 56% seen in male patients. In our study males were found to be 66.5% infected for HCV which is slightly higher than the previous study conducted by Madhavan *et al.* [7]. HCV prevalent studies conducted by several countries have detected HCV antibodies in Hemodialysis patients using ELISA. Moderate sensitivity & high specificity was found to be definite in detecting HCV infection by ELISA. Even though Antibody detection is highly specific, it may fail to detect the early or previous infection of HCV. Therefore, PCR is the only alternate method for detection of early stage of HCV infection and it is most efficient one rather than the antibody detection. Hepatitis C virus can identify the infection in human within 14 days. Although in recovering patients the virus may disappear, whereas in chronic patient's virus may persist for a longer duration. In this scenario, HCV antibody testing & RNA PCR are suggested for detection of HCV. Main drawback of Molecular methods is the cost effective and technical experts to perform the test [12-13]. Statistical studies were carried out by using both Chi-square test & Fisher's exact test to investigate various characteristics associated with the distribution of hemodialysis patients and the predominance of Hepatitis C virus infection. Initially, we analyzed the distribution of patients based on their age, classifying them into distinct age brackets (21-40, 41-60, 61-80, and >80 years). The Chi-square test was used to ascertain whether there were notable disparities in patient distribution among these age categories. In addition, Fisher's exact test was used as a substitute when the expected cell counts were low, assuring the strength and reliability of our findings. Furthermore, we examined the distribution of patients based on gender and the frequencies of HCV positive. In this study, we conducted a comparative analysis across the male & female predominance of HCV-positive individuals.

## Limitations of the study:

A major limitation of our study is the comparatively limited sample size, which limits the applicability of our findings. Moreover, depending solely on data from one tertiary care institution may not provide an accurate representation of wider demographic patterns. In addition, our study did not have sufficient long-term follow-up, which prevented a thorough assessment of the long-term outcomes of patients who tested positive for HCV. This study emphasizes the importance of regular HCV screening in HD patients using RT-PCR due to the limitations of antibody-based detection methods such as ELISA. Healthcare professionals can enhance their management and prevention of HCV infections in HD patients by addressing the identified risk factors and implementing specific interventions.

## Conclusion:

Hepatitis C virus certainly spreads the disease from person to person. Hence, the early identification plays a major role in preventing the cross-infections. Therefore, a rigid universal precaution has to be implemented in dialysis units & also in the isolation area too.

## Figures and Tables

**Table 1:** Age-wise distribution of hemodialysis patients

**Age Groups**	**No. of Patients (n = 92)**	**Percentage (%)**
21-40	29	32
41-60	42	46
61-80	20	22
>80	1	1.08
Total	92	100
N= number of samples; %- Percentage

**Table 2:** Gender wise Distribution of Hemodialysis patients

**Gender**	**HCV Positive**	**HCV Negative**	**Total**	**Positivity (%)**
Male	7	65	70	14
Female	2	20	22	9
Total	9	85	92	9.8
N - Number; %- Percentage; HCV- Hepatitis C virus

**Table 3:** Seroprevalence of HCV in Hemodialysis patients by various diagnostic methods

	**Anti-HCV**			**HCV RNA**		
Total no. of patients	Positive	Negative	Percentage %	Positive	Negative	Percentage %
92	7	85	7.6	9	83	9.8
%- Percentage; N- Number
